# Role of aneuploid circulating tumor cells and CD31^+^ circulating tumor endothelial cells in predicting and monitoring anti‐angiogenic therapy efficacy in advanced NSCLC

**DOI:** 10.1002/1878-0261.13092

**Published:** 2021-09-12

**Authors:** Tongmei Zhang, Lina Zhang, Yuan Gao, Ying Wang, Yanxia Liu, Hongmei Zhang, Qunhui Wang, Fanbin Hu, Jie Li, Jinjing Tan, Daisy Dandan Wang, Olivier Gires, Peter Ping Lin, Baolan Li

**Affiliations:** ^1^ Department of Medical Oncology, Beijing Chest Hospital Capital Medical University Beijing Tuberculosis and Thoracic Tumor Research Institute Beijing China; ^2^ Department of Cellular and Molecular Biology Beijing Chest Hospital, Capital Medical University Beijing Tuberculosis and Thoracic Tumor Research Institute Beijing China; ^3^ Cytelligen San Diego CA USA; ^4^ Department of Otorhinolaryngology, Head and Neck Surgery, University Hospital LMU Munich Germany

**Keywords:** bevacizumab, EMT and EndoMT, EpCAM and vimentin, prognosticators, SE‐iFISH, therapy efficacy

## Abstract

Prognosticating the efficacy of anti‐angiogenic therapy through longitudinal monitoring and early detection of treatment resistance in cancer patients remain highly challenging. In this study, co‐detection and comprehensive phenotypic and karyotypic molecular characterization of aneuploid circulating tumor cells (CTCs) and circulating tumor endothelial cells (CTECs) were conducted on non‐small cell lung cancer (NSCLC) patients receiving bevacizumab plus chemotherapy. Prognostic values of the cell‐based significant univariate risk factors identified by Cox regression analyses were progressively investigated. Subjects showing an increase in total post‐therapeutic platelet endothelial cell adhesion molecule‐1 (CD31)^–^ CTCs and CD31^+^ CTECs exhibited a significantly reduced median progression‐free survival (mPFS) and overall survival. Further stratification analyses indicated that pretherapeutic patients bearing vimentin (Vim)^+^ CTECs (mesenchymal M‐type) at baseline revealed a significantly shortened mPFS compared with patients with Vim^–^ CTECs. Post‐therapeutic patients harboring epithelial cell adhesion molecule (EpCAM)^+^ CTCs and CTECs (epithelial E‐type), regardless of Vim expression or not, showed a significantly reduced mPFS. Post‐therapeutic patients possessing *de novo* EpCAM^+^/Vim^+^ (hybrid E/M‐type) CTECs displayed the shortest mPFS. Patients harboring either pre‐ or post‐therapeutic EpCAM^–^/Vim^–^ null CTECs (N‐type) exhibited a better response to therapy compared to patients harboring EpCAM^+^ and/or Vim^+^ CTECs. The presented results support the notion that baseline Vim^+^ CTECs and post‐therapeutic EpCAM^+^ CTCs and CTECs are predictive biomarkers for longitudinal monitoring of response to anti‐angiogenesis combination regimens in NSCLC patients.

## Graphical abstract

Aneuploid CD31^−^ CTCs and CD31^+^ CTECs, a pair of mutually related and interacting cellular circulating tumor biomarkers, play a critical role in tumorigenesis, neovascularization, and cancer metastasis. Longitudinally comprehensive co‐detection of diverse subtypes of CTCs and CTECs during therapy by iFISH may effectively prognosticate and real‐time monitor therapeutic efficacy or emerging resistance in cancer patients subjected to anti‐angiogenic combination regimens.

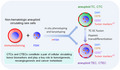

**Abbreviations**
CTC: circulating tumor cell
CTEC: circulating tumor endothelial cell
EMT: epithelial‐to‐mesenchymal transition
EndoMT: endothelial‐to‐mesenchymal transition
iFISH: immunostaining fluorescence *in situ* hybridization
mets: metastases
TEM: tumor endothelial cell‐specific marker

## 1 Introduction

Tumor neovascularization is primarily composed of endothelium‐dependent angiogenesis and vasculogenesis [1]. Neoangiogenesis, a hallmark of neoplasms, is essential for tumorigenesis, progression, and metastasis formation [2]. In tumor neovasculature, endothelial cells (ECs) constitute the lining of the blood and lymphatic vessels. Vascular endothelial growth factor (VEGF), one of the key regulators of angiogenesis, is highly expressed by many carcinoma cells including lung cancer cells. In the hypoxic tumor microenvironment (TME), VEGF is secreted from neoplastic cells, followed by its binding to the VEGF receptor (VEGFR) expressed on ECs [3], thereby promoting tumor angiogenesis.

Endothelial cells in tumor vasculature are known as tumor ECs (TECs) [4, 5], showing cytogenetic abnormalities of chromosomal aneuploidy and abundant expression of CD31 (platelet endothelial cell adhesion molecule‐1, PECAM‐1) [5]. Aneuploid TECs are predominately derived from the endothelialization of cancer cells and cancerization of ECs induced by hypoxia in the TME [6]. The former process consists of both trans‐differentiation of tumor cells into TECs and heterotypic cell fusion of neoplastic cells with ECs [7, 8]. The contribution of TECs to tumor progression has been recently highlighted [9]. In particular, gene expression landscape profiling performed by the single‐cell RNA sequencing analysis indicated that distinct subpopulations of NSCLC TECs possessing diverse phenotypes are relevant to patients’ survival, VEGF blockade, and regulating immune surveillance, respectively [10]. Similar to CTCs, TECs shed from neoplastic vasculature into the peripheral circulation and turn into aneuploid circulating TECs (namely CTECs) in carcinoma patients [6, 11, 12]. Some tumor endothelial cell‐specific markers (TEMs) are expressed on TECs or CTECs [12, 13]. The clinical significance of CTECs in multiple types of cancers has been recently addressed [12, 14, 15, 16, 17, 18]. CTECs were found to correlate with neoadjuvant chemotherapeutic efficacy in breast cancer patients [15] and immunotherapeutic resistance in lung cancer patients [17].

Both epithelial‐to‐mesenchymal transition (EMT) and endothelial‐to‐mesenchymal transition (EndoMT) [19] are centrally instrumental in tumorigenesis, neovascularization [1], formation of TECs and CTECs [6], as well as cancer metastasis [20, 21, 22]. EpCAM and vimentin, two prototypic epithelial and mesenchymal markers in EMT and EndoMT [20, 23, 24], are of particular clinical values in cancer patients. By virtue of expressing EpCAM [20, 21], CTCs are predictive of poor outcome [25] and are preferentially involved in the formation of lung metastasis in breast cancer patients [26] as well as postsurgical recurrence in hepatocellular carcinoma (HCC) patients [27]. Vimentin, in various primary epithelial cancer cells and CTCs, is regarded as an accelerator for tumor progression and metastasis, and an independent marker for poor prognosis and survival [28, 29].

Bevacizumab (Avastin^®^), the only approved monoclonal antibody for anti‐angiogenic therapy in first‐line treatment of eligible advanced lung cancer patients [30], targets VEGF‐A and sterically disrupts VEGF binding to its receptor expressed on ECs, thereby abolishing VEGF’s angiogenic activity [31]. Currently, apart from clinical and histopathological criteria, there is no valid predictive biomarker suitable for preselecting eligible subjects or timely evaluating the therapeutic efficacy of anti‐angiogenic agents [32]. Additionally, accumulated evidence has indicated that a substantial proportion of neoplasms had either inherent or acquired resistance to bevacizumab during VEGF blockade targeted therapy, which has significantly undermined the clinical application of anti‐angiogenic regimens [3]. It is therefore imperative to establish robust biomarkers with respect to risk stratification, identifying eligible patients, predicting and effectively evaluating clinical response as well as detecting emerging resistance to anti‐angiogenic therapy in real time.

Endothelial cells in circulation were thought to represent a marker of vascular remodeling and active turnover [33]. Attempts to evaluate bevacizumab’s efficacy in a variety of cancer patients via enumeration of overall ECs in peripheral blood have been reported by others [3]. However, the results obtained so far correlating quantitative variations in total CD31^+^ ECs with patients’ response to bevacizumab are conflicting [34], partially due to the existence of a substantial amount of nonmalignance‐related ECs in cancer patients’ peripheral circulation [12]. Moreover, circulating ECs were rarely co‐probed with CTCs to estimate anti‐angiogenic therapy efficacy [35].

In the present study, extending beyond our previous investigation on lung cancer PD‐L1^+^ CTCs and CTECs [17], we took advantage of the EpCAM‐independent subtraction enrichment (SE) strategy [11, 26, 36] to enrich heterogeneously sized nonhematologic circulating rare cells in non‐small cell lung cancer (NSCLC) patients, followed by comprehensive phenotypic and karyotypic molecular characterization of CD31^−^ CTCs and CD31^+^ CTECs performed by the integrated immunostaining fluorescence *in situ* hybridization (iFISH) [17, 37]. In light of the truth that small and large CTCs respectively possess diverse chemotherapy‐resistance mechanisms [38], the potential prognostic value of the specific subtypes of heterogeneously sized EpCAM^+^ and/or vimentin^+^ aneuploid CTCs and CTECs was analyzed with regard to predicting and timely monitoring therapeutic efficacy or emerging resistance in NSCLC patients subjected to anti‐angiogenic combination therapy.

## 2 Methods

### 2.1 Patient enrollment and specimen collection

**Fig. 1:**
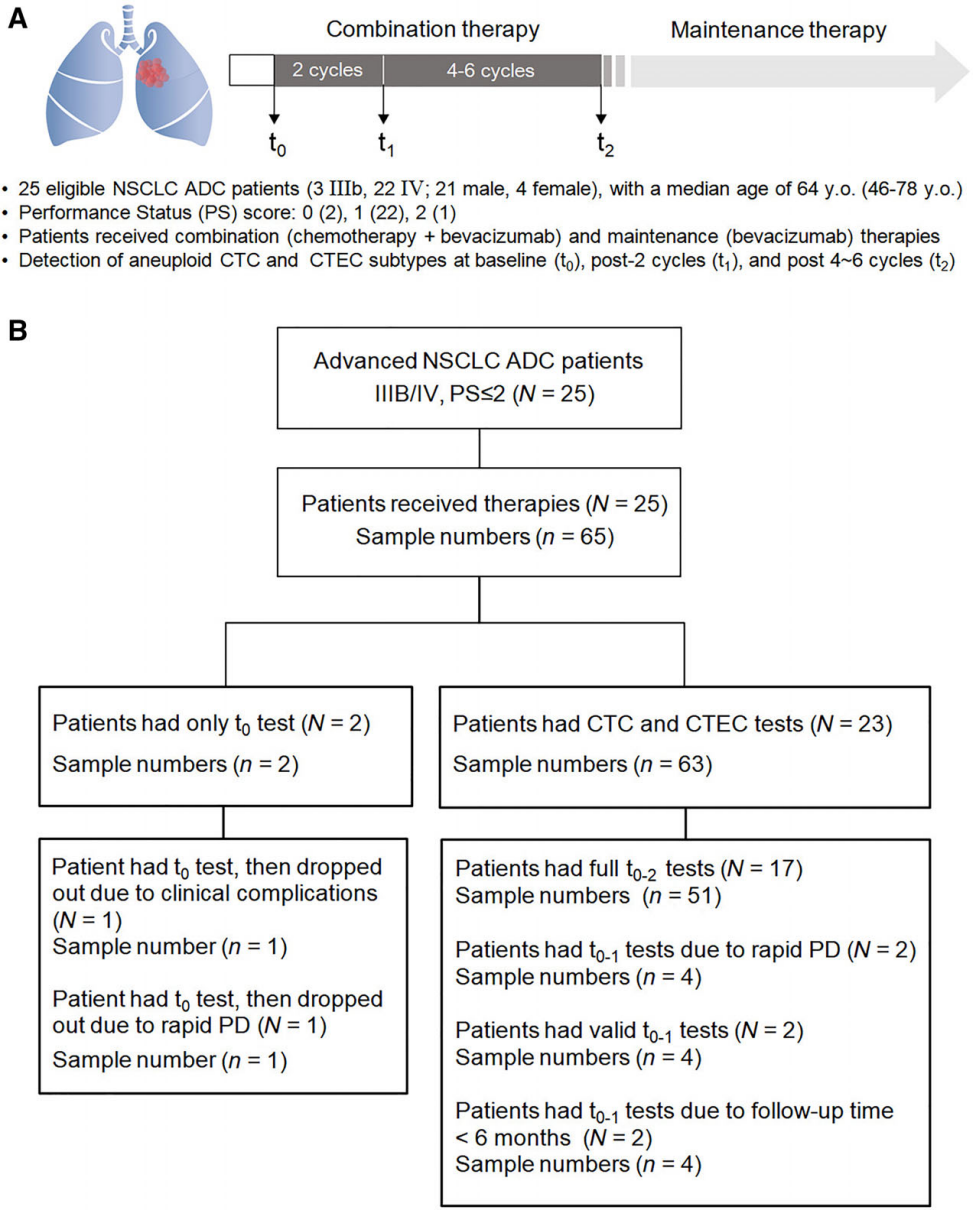
Characteristics of the enrolled NSCLC patients. (A) Characteristics of patients and defined time intervals of CTC and CTEC assessment throughout combination therapy. The recruited 25 treatment‐naive advanced NSCLC adenocarcinoma (ADC) patients were subjected to first‐line combination regimen of platinum‐based chemotherapy and anti‐angiogenic bevacizumab for up to six cycles, followed by maintenance therapy composed of bevacizumab treatment. Detection of CTCs and CTECs was performed at the indicated time points of *t*_0_ (baseline), *t*_1_ (postcombination therapy, 2 cycles), and *t*_2_ (postcombination therapy, 4–6 cycles). (B) Quantitative illustration of patients and specimens throughout therapy. A total of 65 clinical samples in 25 patients were collected for assessment of overall CTCs and CTECs as well as their subtypes. A total of 21 patients are eligible for the follow‐up survival study.

As illustrated in Fig. 1A, a total of 25 eligible NSCLC adenocarcinoma (ADC) patients, including three‐stage IIIB and 22 stage IV subjects, were prospectively enrolled following the NCCN guidelines from November 2017 to July 2019. All recruited treatment‐naive patients had a performance status (PS) score ≤ 2. Three enrolled IIIB patients, not suitable for concurrent chemotherapy but eligible for receiving the same combination regimen administered to stage IV patients, were treated with platinum‐based chemotherapy plus anti‐angiogenic bevacizumab according to the National Comprehensive Cancer Network (NCCN) Clinical Guidelines 8.2020 Non‐Small Cell Lung Cancer. Patients were subjected to four‐to‐six cycles of the combination therapy. Clinical responses were evaluated once in every two treatment cycles by computed tomography (CT) scanning according to the Response Evaluation Criteria in Solid Tumors (recist, version 1.1) (National Cancer Institute, Bethesda, MD, USA). Stable disease (SD), partial response (PR), or complete response (CR) patients subsequently received maintenance therapy of bevacizumab alone until disease progression (PD), attainment of unacceptable toxicity, or patient death. Follow‐up of patients was terminated in February 2020.

Blood samples were periodically collected from patients at baseline (*t*_0_), post‐two (*t*_1_), and post‐four‐to‐six treatment cycles (*t*_2_) (Fig. 1A). Scheduled assessments for some patients were unavailable due to unforeseeable clinical complications (Fig. 1B). A total of 23 clinically eligible patients were assessed at *t*_0_ to *t*_1_ (*t*_0‐1_) and 17 of them had full tests from *t*_0_ to *t*_2_ (*t*_0‐2_). Follow‐up information was available for a total of 21 subjects.

Consent forms signed by all subjects were approved by the Ethics Review Committees (ERC) of Beijing Chest Hospital, Capital Medical University, Beijing, China. The written consent forms were received from each patient prior to blood collection. The clinical study was performed according to the Declaration of Helsinki Principles.

### 2.2 SE‐iFISH

SE‐iFISH protocol (Cytelligen, San Diego, CA, USA) was similar to that previously published with minor modifications [11]. With respect to subtraction enrichment (SE), briefly, six milliliters of blood was centrifuged to separate plasma. Sedimented blood cells were resuspended with three ml hCTC buffer and loaded on the nonhematologic cell separation matrix. Samples were centrifuged, followed by the collection of the solution above red blood cells (RBCs). Solution containing WBCs was incubated with magnetic beads conjugated to a cocktail of antileukocyte mAbs. WBCs‐bound immuno‐beads were subsequently removed. The remaining nonhematologic cells were mixed with cell fixative, then smeared on formatted CTC slides, and dried for subsequent iFISH processing.

In regard to iFISH, dried monolayer cells on the coated CTC slides were hybridized with centromere probe 8 (CEP8) SpectrumOrange (Vysis, Abbott Laboratories, Chicago, IL, USA), which has been approved by the USFDA to identify aneuploid solid tumor cells. Samples were subsequently incubated with the indicated monoclonal antibodies, including Alexa Fluor (AF)594‐anti‐CD45 (Clone 9.4), AF488‐anti‐EpCAM (Clone 9C4), Cy5‐anti‐CD31(Clone WM59), and Cy7‐anti‐vimentin (Clone 1D3) [11, 39]. Conjugation of diverse antibodies to each specific fluorescent dye was performed at Cytelligen. After washing, samples were mounted with mounting media containing DAPI (Vector Laboratories, Burlingame, CA, USA) and subjected to the automated Metafer‐i•FISH^®^ CTC six‐channel 3D scanning and image analyzing system codeveloped by Carl Zeiss (Oberkochen, Germany), MetaSystems (Altlussheim, Germany), and Cytelligen [11]. Identification criteria for the maximized six‐color CD31^−^ CTCs include the following: DAPI^+^/CD45^−^/CD31^−^/EpCAM^+/−^/vimentin^+/−^ aneuploid cells (CEP 8) and DAPI^+^/CD45^−^/CD31^−^/EpCAM^+^, or vimentin^+^ near‐diploid cells [40, 41]; criteria for CD31^+^ CTECs include the following: DAPI^+^/CD45^−^/CD31^+^/EpCAM^+/−^/vimentin^+/−^ aneuploid cells and DAPI^+^/CD45^−^/CD31^+^/EpCAM^+^, or vimentin^+^ near‐diploid cells.

### 2.3 Statistical analyses

All statistical analyses were performed with ibm spss statistics 25.0 (Armonk, NY, USA). Chi‐squared tests were applied to compare categorical data. Significant univariable risk factors were identified by Cox proportional hazards regression model analyses. Positive correlation of CTCs and CTECs expressing EpCAM and/or vimentin with therapeutic efficacy was analyzed using Fisher’s exact test. A one‐way analysis of variance (ANOVA) was applied to analyze the difference among three groups of separate data, including *t*_0_, *t*_1,_ and *t*_2_ values of total CTCs or CTECs in the ascending or descending cohort of patients. Kaplan–Meier survival curves of progression‐free survival (PFS) and overall survival (OS) were created based upon the numbers of patients in the ascending or descending cohort. Log‐rank and Breslow tests were applied to compare the survival curves. All the *P* values are two‐sided. *P < 0.05, **P < 0.01, and ***P < 0.001 are considered statistically significant, very significant, and extremely significant. PFS and OS are defined as the duration from initial blood collection to the date of disease progression (enlarged primary lesion, intrapulmonary or distant metastasis) and patient’s death, respectively. Sankey diagrams were plotted utilizing the rstudio software v8.10 (Boston, MA, USA).

## 3 Results

### 3.1 Quantification of aneuploid CTCs and CTECs co‐detected by iFISH

**Fig. 2:**
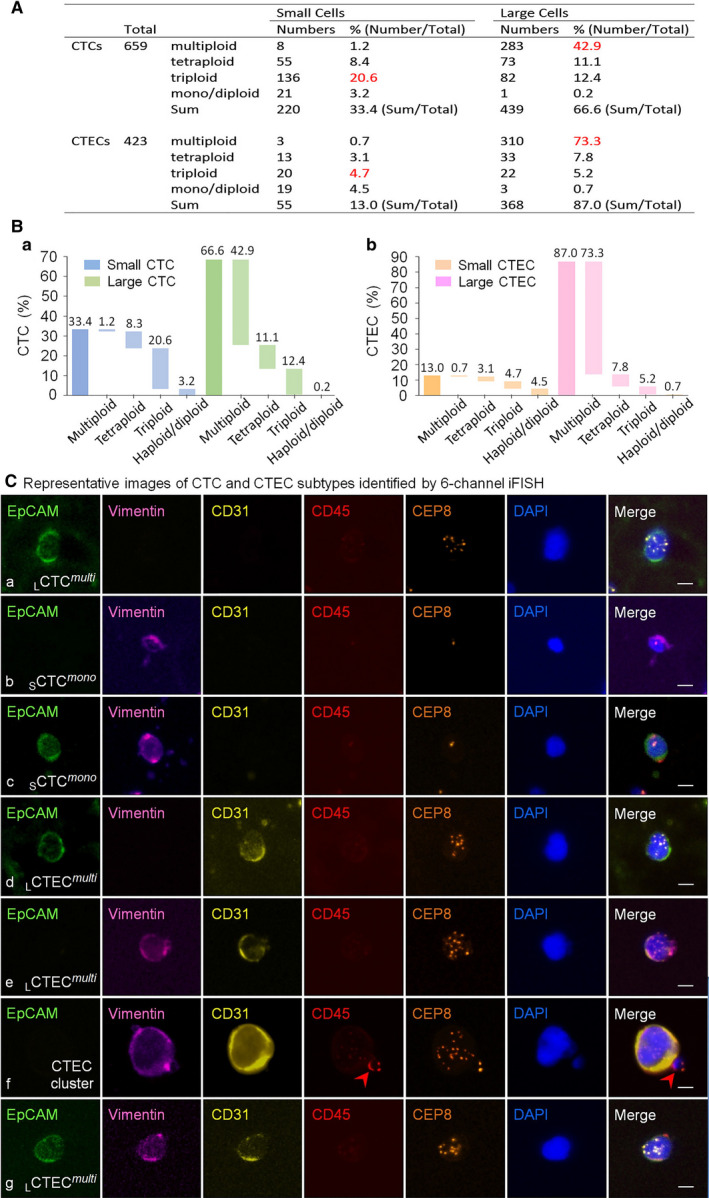
Quantification and molecular characterization of co‐detected diverse subtypes of aneuploid CTCs and CTECs. (A) Quantitative analysis of molecularly characterized CTCs and CTECs in different cell sizes. *CTCs*: among 659 CTCs, 220 of them are small cell sized _S_CTCs (220 out of 659, 33.4%) with 20.6% (136 out of 659) being triploid (_S_CTCs*^tri^*); remaining 439 CTCs are large _L_CTCs (439 out of 659, 66.6%) with 42.9% (283 out of 659) being multiploid (_L_CTCs*^multi^*). *CTECs*: out of 423 CTECs, 55 are _S_CTECs (55 out of 423, 13%) with 4.7% (20 out of 423) being triploid (_S_CTECs*^tri^*); the rest of 368 cells are _L_CTECs (368 out of 423, 87%) with 73.3% (310 out of 423) being multiploid (_L_CTECs*^multi^*). Highest percentages of different subtypes are indicated in red font. (B) Compositional waterfall map: compositions of CTC (Ba) and CTEC subtypes (Bb) are depicted in a schematic waterfall map. Percentages of each subtype as described in (A) are obtained from quantification analysis of total CTCs and CTECs longitudinally detected throughout therapy and indicated on the top of each column. (C) Representative images of CTC and CTEC subtypes identified by iFISH. (C‐a) A representative image of a large multiploid CTC (_L_CTC*^multi^*) expressing EpCAM (EpCAM^+^/vimentin (Vim)^−^/CD31^−^, epithelial E‐type). (C‐b) A representative image of a haploid mesenchymal small CTC (_S_CTC*^mono^*) with an EpCAM^−^/Vim^+^/CD31^−^ phenotype (mesenchymal M‐type). (C‐c) A representative image of a haploid small CTC (_S_CTC*^mono^*) expressing both EpCAM and vimentin (EpCAM^+^/Vim^+^/CD31^−^, intermediate hybrid E/M‐type). (C‐d) A representative image of a large multiploid E‐type CTEC (_L_CTEC*^multi^*, EpCAM^+^/Vim^−^/CD31^+^). (C‐e) A representative image of a large multiploid M‐type CTEC (_L_CTEC*^multi^*, EpCAM^−^/Vim^+^/CD31^+^). (C‐f) A representative image of a M‐type CTEC fusion cluster with multinuclei (EpCAM^−^/Vim^+^/CD31^+^) and a diploid CD45^+^ WBC attached (red arrow). (C‐g) A representative image of a large multiploid E/M‐type CTEC (_L_CTEC*^multi^*, EpCAM^+^/Vim^+^/CD31^+^). All the representative images are from the image library of all patients’ CTCs and CTECs longitudinally detected throughout therapy as described in (A). Bars, 5 µm.

Six‐channel iFISH was applied to perform a phenotypic and karyotypic characterization of different subtypes of aneuploid CTCs and CTECs enriched from NSCLC patients (see Fig. 1 for a schematic representation of the study protocol). Diverse subtypes of CTCs and CD31^+^ CTECs, classified upon cell size, degree of aneuploidy, and expression of EpCAM or vimentin, were observed in patients throughout therapy. Quantitative analysis of heterogeneously sized aneuploid CTCs and CTECs in 65 blood specimens of 25 patients is illustrated in Fig. 2A and the compositional waterfall map in Fig. 2B. As revealed in Fig. 2A, among a total of 659 CTCs enriched from the totality of blood samples, there were 220 small (≤ 5 µm WBC, 220/659 = 33.4%, blue in Fig. 2Ba) and 439 large cells ( 5 µm WBC, 439/659 = 66.6%, green in Fig. 2Ba). Trisomy 8 was the main karyotype for small CTCs (_S_CTCs*^tri^*, 136 cells, 136/659 = 20.6%, blue), whereas multiploidy (≥ pentasomy 8) constituted the principal karyotype for large CTCs (_L_CTCs*^multi^*, 283 cells, 283/659 = 42.9%, green). The totality of 423 CTECs consisted of 55 small (55/423 = 13%, orange in Fig. 2Bb) and 368 large cells (368/423 = 87%, pink in Fig. 2Bb). Most large CTECs were multiploid (_L_CTECs*^multi^*, 310 cells, 310/423 = 73.3%, pink), whereas small CTECs were heterogeneous in varieties of aneuploidy degrees of chr8 from monosomy, disomy/near‐disomy [40, 41] to trisomy 8 (orange). Further numerical analysis of aneuploid CTC and CTEC subtypes expressing EpCAM or vimentin detected during therapy is described below in Comprehensive analysis of CTCs and CTECs in patient cohorts.

Representative images of CTCs or CTECs identified by iFISH are illustrated in Fig. 2C. As shown in Fig. 2Ca‐c, CTCs reveal different degrees of aneuploidy, heterogeneous morphologies, and phenotypes including large multiploid (≥ pentasomy 8) EpCAM^+^/Vim^−^/CD31^−^ (epithelial E‐type) CTCs (_L_CTC*^multi^*, Fig. 2Ca) [26], small EpCAM^−^/Vim^+^/CD31^−^ (mesenchymal M‐type) haploid CTCs (_S_CTC*^mono^*, Fig. 2Cb), and a haploid EpCAM^+^/Vim^+^/CD31^−^ CTC (intermediate hybrid E/M‐type) (Fig. 2Cc) [42]. Large multiploid CTECs (_L_CTEC*^multi^*) with phenotypes of EpCAM^+^/Vim^−^/CD31^+^ (E‐type) and EpCAM^−^/Vim^+^/CD31^+^ (M‐type) are shown in Fig. 2Cd‐e, respectively. Figure 2Cf reveals an EpCAM^−^/Vim^+^/CD31^+^ (M‐type) fusogenic CTEC cluster consisting of two cells with a bound CD45^+^ WBC. WBCs attached to CTCs were reported to accelerate cancer cells’ metastatic potential [43]. An EpCAM^+^/Vim^+^/CD31^+^ (E/M‐type) multiploid CTEC is shown in Fig. 2Cg.

### 3.2 Comprehensive analysis of CTCs and CTECs in patient cohorts

**Fig. 3:**
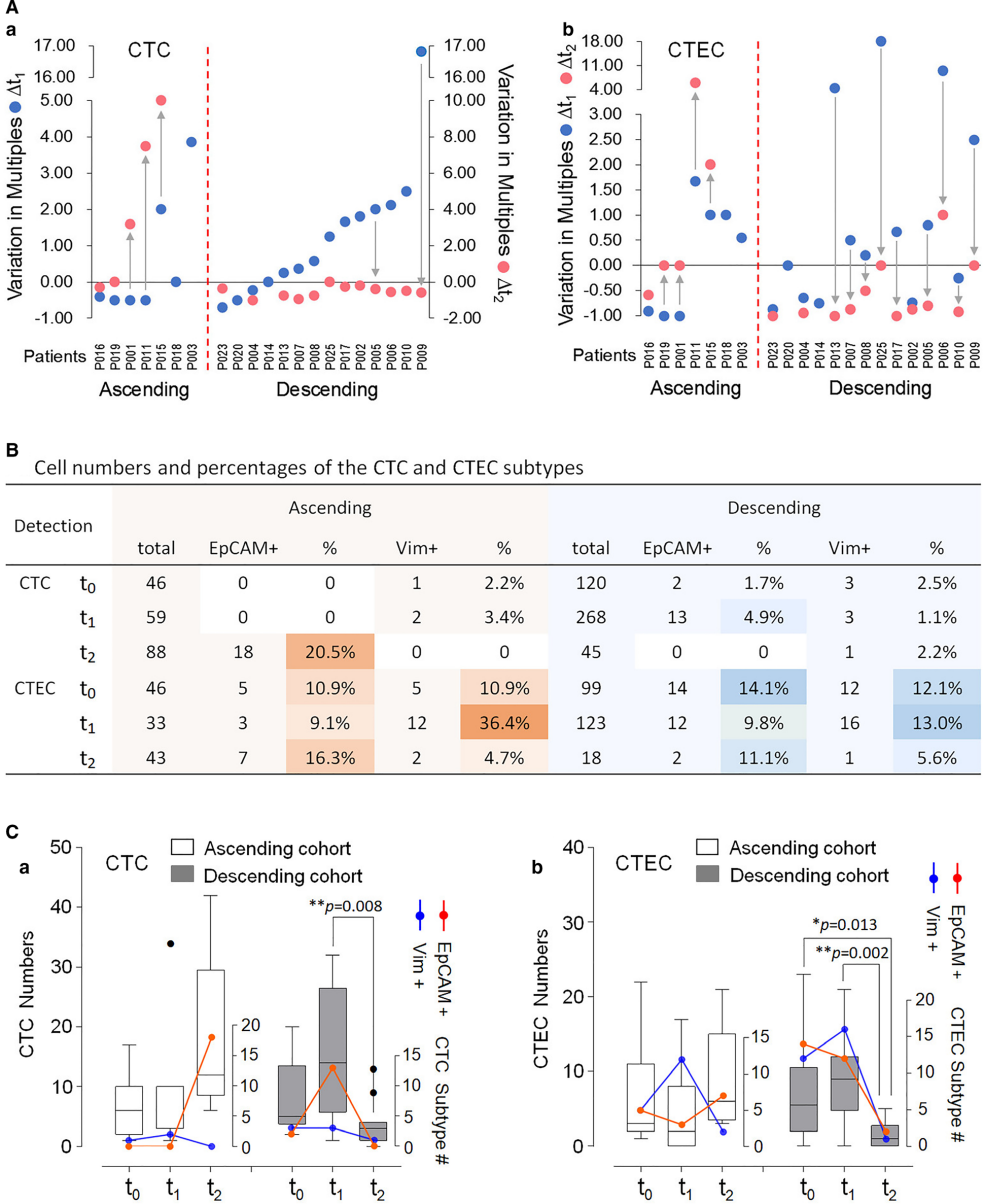
Comprehensive analysis of heterogeneous‐sized CTCs and CTECs. (A) Categorization of patients. Based upon quantitative variation of CTCs (A‐a) and CTECs (A‐b) throughout therapy, patients are categorized into ascending (∆*t*_2_ red dot > ∆*t*_1_ blue dot) and descending (∆*t*_2_ < ∆*t*_1_) cohorts which are divided by a red dashed line. In each cohort, CTCs and CTECs exhibit a similar variation trend. (B) Heattable: longitudinal variation in cell numbers and percentages of the specific CTC and CTEC subtypes. Variation of the percentage of CTC or CTEC subtypes during treatment is similar to the cell number change in EpCAM^+^ or Vim^+^ CTCs and CTECs detected from *t*_0_ to *t*_2_. (C) Quantitative variation of CTCs and CTECs during therapy. (C‐a) CTC. In the ascending cohort (white bars), compared to the baseline median value (6 cells, *t*_0_), the post‐therapeutic median values of total CTCs exhibit a downward‐upward variation pattern of 3 cells at *t*_1_ and 12 cells at *t*_2_ (left *y*‐axis). Number of EpCAM^+^ CTCs (red) increased from 0 (*t*_0‐1_) to 18 cells (*t*_2_) (right *Y*‐axis). In the descending cohort, the median values of total CTCs (gray bars, 5 cells at *t*_0_) display an upward‐downward variation pattern, showing 14 cells at *t*_1_ and 3 cells at *t*_2_, **P = 0.008 (*t*_1_ vs *t*_2_). EpCAM^+^ CTCs have the same upward‐downward pattern (red): 2 (*t*_0_), 13 (*t*_1_), and 0 cell (*t*_2_). Black dots: discrete data. (C‐b) CTEC. Total and EpCAM^+^ CTEC number in both ascending and descending cohorts respectively display similar downward‐upward and upward‐downward variation patterns. Differences in the median values of total CTECs are statistically significant, *P = 0.013 (*t*_0_ vs *t*_2_), and **P = 0.002 (*t*_1_ vs *t*_2_), log‐rank test. Quantitative changes in Vim^+^ CTCs or CTECs following therapy (blue) reveal an upward (*t*_1_)‐downward (*t*_2_) pattern in most cases.

Quantitative variations in CTCs, CTECs, and their subtypes expressing EpCAM or vimentin in patients throughout therapy were analyzed. Changes in CTC and CTEC numbers were analyzed by comparison of ∆*t*_1_ = (*t*_1_ − *t*_0_)/*t*_0_ vs ∆*t*_2_ = (*t*_2_ − *t*_0_)/*t*_0_. Among 23 patients, two subjects were excluded for follow‐up analysis because follow‐up time was < 6 months (Fig. 1B). There was a total of 59 blood samples in remaining 21 patients. Exact cell numbers and values of ∆*t*_1_ and ∆*t*_2_ for each patient are described in Table 1. A total of 21 patients were categorized into ascending (∆*t*_2_ > ∆*t*_1_, 7 patients) and descending (∆*t*_2_ < ∆*t*_1_, 14 patients) cohorts, respectively. Subjects who did not have *t*_2_ values available were classified upon *t*_1_ > *t*_0_ (ascending) or *t*_1_ < *t*_0_ (descending) for CTCs or CTECs. As graphically depicted in Fig. 3Aa‐b, CTCs (Aa) and CTECs (Ab) in the ascending or descending cohort, divided by a red dashed line, exhibited a similar variation pattern in sync with combination therapy.

**Table 1:** Quantitative analysis of CTCs and CTECs in both ascending and descending cohorts. Variation in multiples: ∆*t*_1_ = (*t*_1_ − *t*_0_)/*t*_0_, ∆*t*_2_ = (*t*_2_ − *t*_0_)/*t*_0_. *t*_0_, cell numbers at baseline; *t*_1_, cell numbers at 2 cycles; *t*_2_, cell numbers at 4 ~ 6 cycles; n/a, not available. Ascending cohort: ∆*t*_2_ > ∆*t*_1_, or *t*_1_ > *t*_0_ if *t*_2_ is not available; descending cohort: ∆*t*_2_ < ∆*t*_1_, or *t*_1_ < *t*_0_ if *t*_2_ is not available.

Patients	CTCs					CTECs				
	*t*_0_	*t*_1_	∆*t*_1_	*t*_2_	∆*t*_2_	*t*_0_	*t*_1_	∆*t*_1_	*t*_2_	∆*t*_2_
	Ascending									
P016	17	10	−0.41	12	−0.29	22	2	−0.91	9	−0.59
P019	6	3	−0.50	6	0	3	0	−1.00	3	0
P001	10	5	−0.50	42	3.20	4	0	−1.00	4	0
P011	2	1	−0.50	17	7.50	3	8	1.67	21	6.00
P015	1	3	2.00	11	10.00	2	4	1.00	6	2.00
P018	3	3	0	n/a	n/a	1	2	1.00	n/a	n/a
P003	7	34	3.86	n/a	n/a	11	17	0.55	n/a	n/a
	Descending									
P023	20	6	−0.70	13	−0.35	8	1	−0.88	0	−1.00
P020	2	1	−0.50	n/a	n/a	0	0	0	n/a	n/a
P004	18	14	−0.22	0	−1.00	17	6	−0.65	1	−0.94
P014	3	3	0	n/a	n/a	4	1	−0.75	n/a	n/a
P013	4	5	0.25	1	−0.75	2	11	0.45	0	−1.00
P007	19	26	0.37	1	−0.95	8	12	0.50	1	−0.88
P008	12	19	0.58	3	−0.75	10	12	0.20	5	−0.50
P025	4	9	1.25	4	0	0	18	18.00	0	0
P017	12	32	1.67	9	−0.25	6	10	0.67	0	−1.00
P002	5	14	1.80	4	−0.20	23	6	−0.74	3	−0.87
P005	5	15	2.00	3	−0.40	5	9	0.80	1	−0.80
P006	9	28	2.11	4	−0.56	2	21	9.50	4	1.00
P010	2	7	2.50	1	−0.50	12	9	−2.50	1	−0.92
P009	5	89	16.80	2	−0.60	2	7	2.50	2	0

Longitudinal variation of cell numbers and percentages of the specific subtype of CTC and CTEC expressing EpCAM or vimentin in both ascending and descending cohort are displayed in the heattable in Fig. 3B.

Further analysis of the total numbers of CTCs, CTECs, and their subtypes is depicted in Fig. 3Ca‐b. As revealed in Fig. 3Ca, the median values of total CTCs (including all the EpCAM or Vim^+ and –^ CTCs) in the ascending cohort were 6 (Min 1/Max 17, *t*_0_), 3 (Min 1/Max 34, *t*_1_), and 12 cells (Min 6/Max 42, *t*_2_), respectively. Although median values of CTCs in this cohort did not show a statistically significant change (*P* = 0.210), they all had the same upward variation trend in the ascending cohort of patients. Among all CTCs in the ascending cohort, the number of EpCAM^+^ CTCs (red) considerably increased from 0 (*t*_0‐1_) to 18 cells (*t*_2_). Regarding the descending cohort in Fig. 3Ca, the median values of overall CTCs were 5 (Min 2/Max 20, *t*_0_), 14 (Min 1/Max 89, *t*_1_), and 3 cells (Min 0/Max 13, *t*_2_). The difference in median values of total CTCs between *t*_1_ and *t*_2_ was statistically significant (**P = 0.008). EpCAM^+^ CTCs (red) in the descending cohort showed a similar variation pattern to that of overall CTCs, increasing from 2 (*t*_0_) to 13 cells (*t*_1_), then decreasing to 0 (*t*_2_). Vim^+^ CTCs in the same cohort (blue) decreased from 3 cells (*t*_0‐1_) to 1 cell (*t*_2_).

As shown in Fig. 3Cb, median values of overall CTECs in the ascending cohort were 3 (Min 1/Max 22, *t*_0_), 2 (Min 0/Max 17, *t*_1_), and 6 cells (Min 3/Max 21, *t*_2_), *P* = 0.642. In the same ascending cohort, an inverse variation pattern was found in EpCAM^+^ and Vim^+^ CTECs detected from *t*_0_ to *t*_2_ with Vim^+^ CTECs increasing at *t*_1_ and decreasing again at *t*_2_, whereas EpCAM^+^ CTECs slightly decreased at *t*_1_ and increased again at the same time point of *t*_2_. Regarding the descending cohort, the number of total CTECs had a median value of 6 (Min 0/Max 23, *t*_0_), 9 (Min 0/Max 21, *t*_1_), and 1 cell (Min 0/Max 5, *t*_2_), respectively. Unlike CTCs (*P* = 0.210) and CTECs (*P* = 0.642) in the ascending cohort, differences in the median values of total CTECs between *t*_0_ and *t*_2_ (*P = 0.013), and between *t*_1_ and *t*_2_ (**P = 0.002) were statistically significant. Similar to the ascending cohort, the quantitative variation of Vim^+^ CTECs in the descending cohort displayed a similar upward (*t*_1_)‐downward (*t*_2_) pattern showing 12 (*t*_0_), 16 (*t*_1_), and 1 cell (*t*_2_), whereas the amount of EpCAM^+^ CTECs steadily decreased, revealing from 14 (*t*_0_), 12 (*t*_1_), then to 2 cells (*t*_2_). The quantitative variation of the post‐therapeutic EpCAM^+^ CTCs and CTECs was in line with the cell number change in total CTCs and CTECs in both ascending and descending cohorts of patients.

The results of Fig. 3 indicated that Vim^+^ CTECs distinctly exhibited a unified upward (*t*_1_)‐downward (*t*_2_) biphasic response pattern in both ascending and descending cohorts, which was also reported on breast cancer patients receiving neoadjuvant chemotherapy [15]. However, the variation patterns of EpCAM^+^ CTCs and CTECs in both ascending and descending cohorts of NSCLC patients treated with anti‐angiogenic combination regimen consistently matched the total CTCs’ and CTECs’ fluctuation from *t*_0_ to *t*_2_.

As illustrated in Table 2, additional comprehensive morphologic and karyotypic analysis was performed on the specific subtypes of EpCAM^+^ or Vim^+^ CTCs and CTECs shown in Fig. 3Ca‐b. Among a total of 18 EpCAM^+^ CTCs detected at *t*_2_ in the ascending cohort, 14 of them (14/18 = 77.8%) were small cell‐sized _S_CTCs (CD31^−^/CD45^−^) and all 14 (100%) were diploid or near‐diploid (nonaneuploid) _S_CTCs*^di^* [40, 41]. In the descending cohort at *t*_1_, 12 out of 13 (12/13 = 92.3%) EpCAM^+^ CTCs were large cell‐sized _L_CTCs and 11 of them (11/12 = 91.7%) were multiploid _L_CTCs*^multi^*. Regarding CTECs in the ascending cohort, all five baseline (*t*_0_) EpCAM^+^ CTECs were large cells (100%), with four being multiploid _L_CTECs*^multi^* (4/5 = 80%). Four out of five (4/5 = 80%) baseline Vim^+^ CTECs in the ascending cohort were large cells, with 3 of them (3/4 = 75%) being multiploid _L_CTECs*^multi^*. In the same ascending cohort at *t*_1_, nine out of 12 Vim^+^ CTECs (9/12 = 75%) were _s_CTECs and seven of the nine (7/9 = 77.8%) were diploid/near‐diploid _s_CTECs*^di^*, whereas all the particular EpCAM^+^ CTECs (*t*_1_) were _L_CTECs*^multi^* (3/3 = 100%). Detection at *t*_2_ in this cohort showed that all seven EpCAM^+^ CTECs (100%) were _L_CTECs with six of them (6/7 = 85.7%) being _L_CTECs*^multi^*. In the descending cohort, all 14 (100%) baseline EpCAM^+^ CTECs were large cells and 12 of them (12/14 = 85.7%) were _L_CTECs*^multi^*. Among another 12 baseline Vim^+^ CTECs, eight of them (8/12 = 66.7%) were _L_CTECs and all eight (100%) were _L_CTECs*^multi^*. At *t*_1_, all 12 EpCAM^+^ CTECs (100%) were _L_CTECs*^multi^*. Fourteen out of 16 Vim^+^ CTECs at *t*_1_ (14/16 = 87.5%) were _L_CTECs with half of them (7/14 = 50%) being _L_CTECs*^multi^*. Obtained results indicated that in contrast to EpCAM^+^ CTCs heterogeneous in both cell size (small *vs* large) and chromosome ploidy (diploid and multiploid chr8), most EpCAM^+^ CTECs homogeneously exhibited as multiploid large cells (_L_CTECs*^multi^*).

**Table 2:** Compositional analysis of EpCAM^+^ or vimentin^+^ CTCs and CTECs in small (S) and large (L) cell sizes.

	CTC		CTEC		
	Ascending	Descending	Ascending		Descending
	_S_CTCs	_L_CTCs	_S_CTECs	_L_CTECs	_L_CTECs
*t*_0_				EpCAM+ (100%) (multiploid 80%)	EpCAM+ (100%) (multiploid 85.7%)
				Vim+ (80%) (multiploid 75%)	Vim+ (66.7%) (multiploid 100%)
*t*_1_		EpCAM+ (92.3%) (multiploid 91.7%)		EpCAM+ (100%) (multiploid 100%)	EpCAM+ (100%) (multiploid 100%)
			Vim+ (75%) (diploid/near‐diploid 77.8%)		Vim+ (87.5%) (multiploid 50%) (tetraploid 21.4%) (triploid 14.3%)
*t*_2_	EpCAM+ (77.8%) (diploid/near‐diploid 100%)			EpCAM+ (100%) (multiploid 85.7%)	

### 3.3 Prognosis of the ascending and descending cohorts of patients

**Fig. 4:**
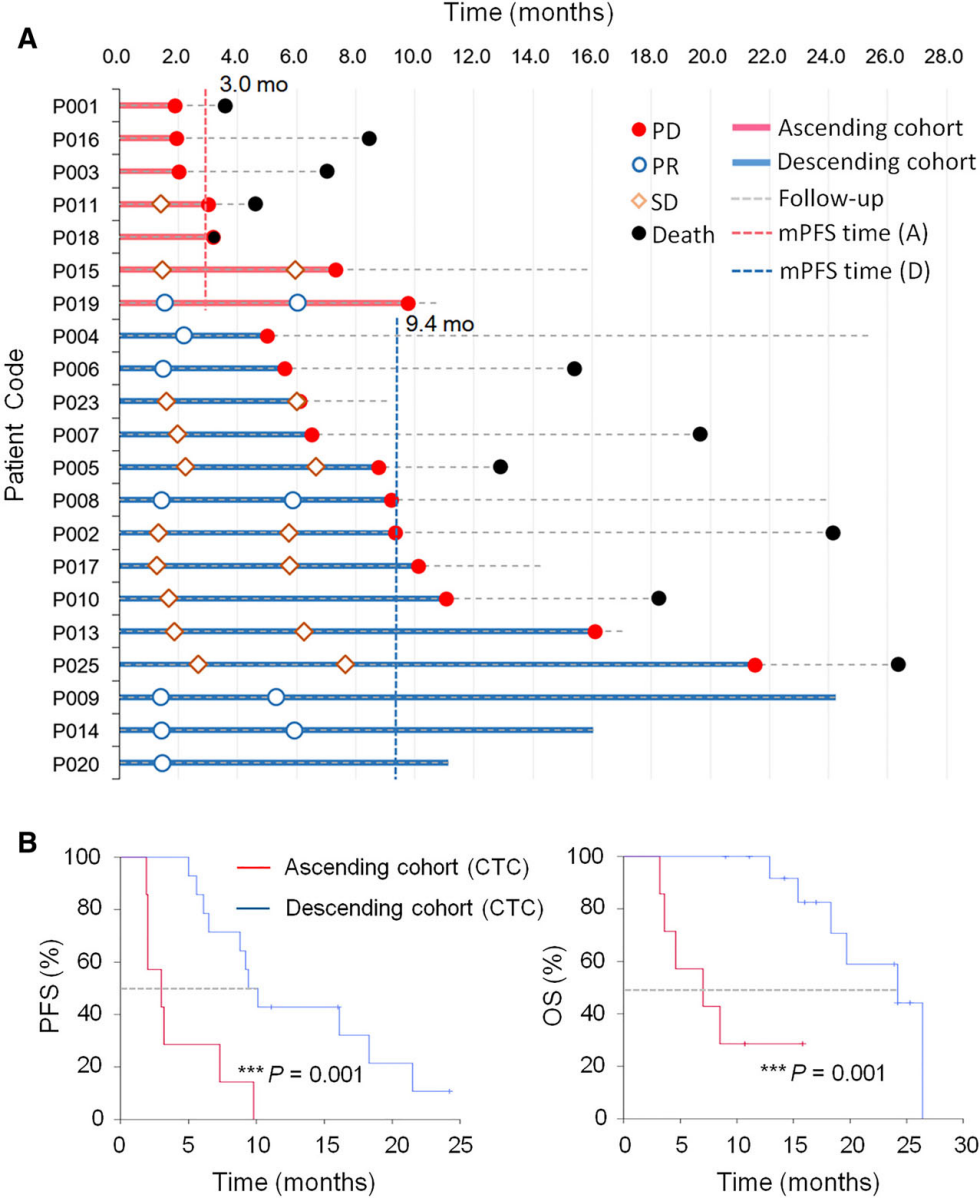
Prognosis analysis of the ascending and descending cohorts of patients. (A) Progressive clinical status of each patient in the ascending (red) and descending (blue) cohorts following therapy is illustrated. (B) Kaplan–Meier survival analysis. Cohorts classified by CTCs. The ascending cohort of patients shows a shortened median progression‐free survival (mPFS) of 3.0 months compared to the prolonged 9.4 months of the descending cohort (***P = 0.001, log‐rank test). Patients in the ascending cohort have a median overall survival (mOS) of 7.0 months, which is significantly shorter than 24.3 months of the descending cohort (***P = 0.001, log‐rank test). The ascending and descending cohorts categorized by CTECs have mPFS and mOS identical to that in cohorts classified by CTCs.

The above analysis illustrates how quantities of CTCs and CTECs as well as cell numbers of their specific subtypes varied in the ascending and descending cohorts. Accordingly, correlation of quantitative variation of CTCs, CTECs, and their various subtypes with patients’ prognosis was analyzed in this section and in Prognostic values of the specific subtypes of EpCAM^+^ and Vim^+^ CTCs and CTECs below, respectively. As revealed in Fig. 4, analysis was performed on 21 follow‐up patients eligible for survival analysis, comprising seven subjects in the ascending cohort (red) and 14 subjects in the descending cohort (blue). Detailed progressive clinical status of each patient throughout therapy is shown in Fig. 4A.

A Cox proportional hazards regression model analysis was performed to identify significant univariable risk factors indicated in red font in Fig. S1. In comparison with risk factors of non‐cell‐based age, gender, and staging, quantitative variation trend of CTCs (*i.e*., ascending *vs* descending) in patients following therapy was the cell‐based significant univariable risk factor, indicating the risk of rapid disease progression (shortened PFS) and rapid patients’ death (reduced OS) for the ascending cohort was 7 (hazard ratio HR = 7.026, 95% CI: 2.081–23.728, ***P = 0.002) and 11.6 (HR = 11.592, 95% CI: 2.082–64.541, ***P = 0.005) times higher than that of descending cohort, respectively. Because ascending and descending cohorts classified by CTC or CTEC variation trend had identical subjects due to the concurrent upward or downward changes in patients (Fig. 3Aa‐b and Table 1), the results of Cox regression analyses performed on the cohorts categorized by CTECs were identical to that of CTCs. Accordingly, subsequent Kaplan–Meier survival analysis was performed on cohorts containing either increasing or decreasing CTCs or CTECs. As depicted in Fig. 4B, the ascending cohort classified by changes of CTC numbers (*N* = 7) had a shorter median PFS (mPFS) of 3.0 months (95% CI: 0.4–5.6 months) compared to 9.4 months (95% CI: 7.8–11.1 months) in the descending cohort (*N* = 14, ***P = 0.001, log‐rank test). Eleven out of 21 subjects were fatal cases in this study. The ascending cohort (*N* = 7) had a median OS (mOS) of 7.0 months (95% CI: 0.8–13.2 months) compared to 24.3 months (95% CI: 13.6–34.8 months) mOS in the descending cohort (*N* = 14, ***P = 0.001, log‐rank test). mPFS and mOS for both CTC‐ and CTEC‐categorized cohorts were identical as explained above. Obtained results indicated that the ascending cohort of patients had a poorer response to bevacizumab in terms of both reduced mPFS and mOS.

### 3.4 Prognostic values of the specific subtypes of EpCAM^+^ and Vim^+^ CTCs and CTECs

The above analysis illustrates how quantitative changes in post‐therapeutic total CTCs and CTECs were relevant to prognosis in both ascending and descending cohorts of patients. Further analysis was performed to investigate whether the existence of the particular subtypes of EpCAM^+^ and Vim^+^ CTCs and CTECs in the same cohort of patients correlated with patients’ response to anti‐angiogenesis regimen.

**Fig. 5:**
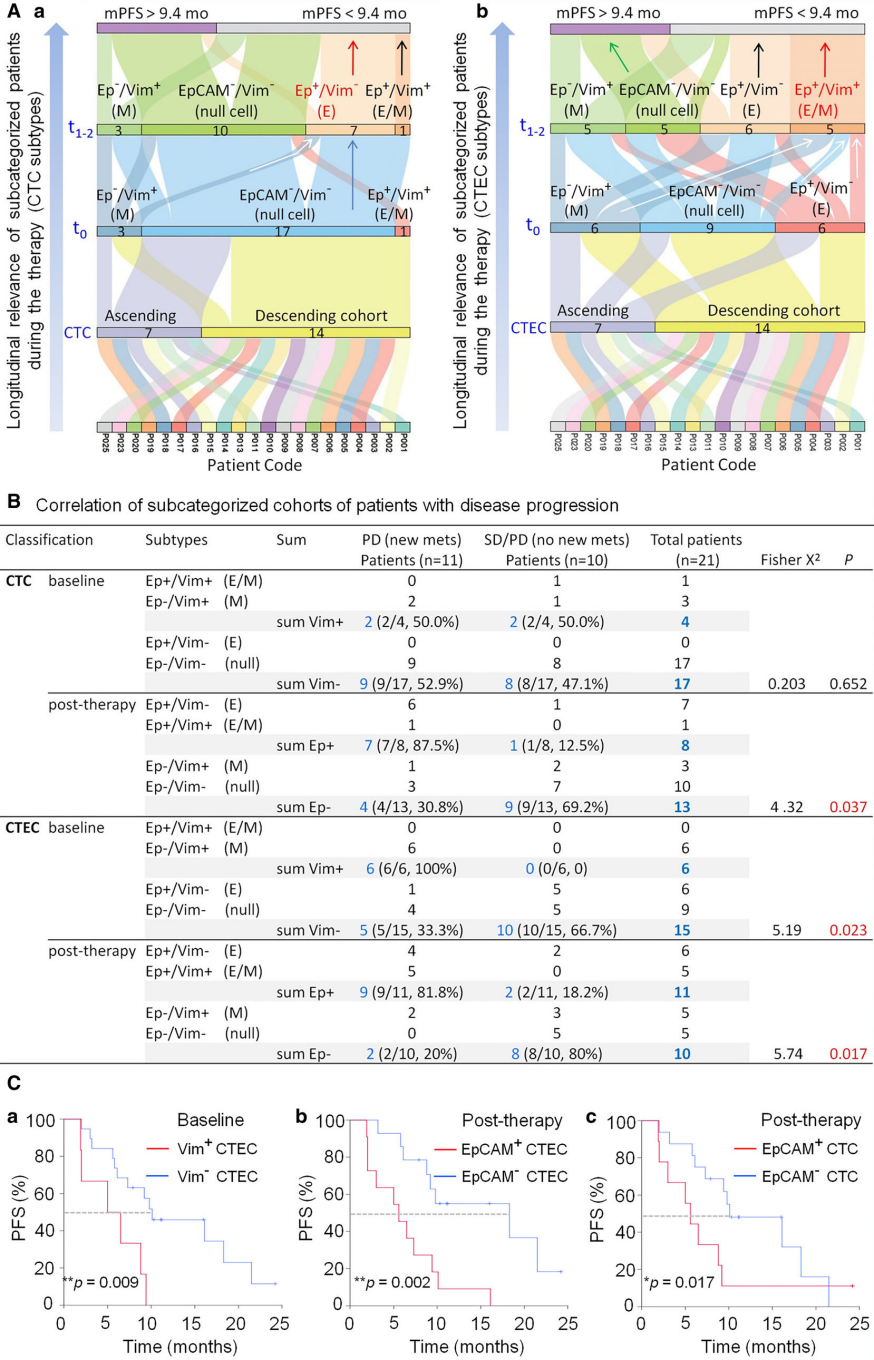
Correlation of aneuploid CTC and CTEC subtypes with poor prognosis. (A) Sankey diagrams: longitudinal analysis of diverse subtypes of CTCs and CTECs detected at the indicated time intervals in a total of 21 patients during therapy. The number of subcategorized patients possessing each subtype of cells is indicated in the figure. (A‐a) CTC. There are three cohorts of patients respectively containing three diverse CTC subtypes at baseline (*t*_0_), including EpCAM^−^/vimentin (Vim)^+^ (M‐type, 3 patients), the most abundant EpCAM^−^/Vim^−^ (nonhematologic aneuploid N‐type null cells, 17 patients), and the least abundant EpCAM^+^/Vim^+^ (hybrid E/M‐type, 1 patient). Following therapy, a *de novo* cohort of patients acquiring EpCAM^+^/Vim^−^ (E‐type) CTCs emerges (red, *t*_1‐2_, 7 patients), with most patients being the pretreatment cohort harboring N‐type null CTCs and a minority being the baseline M‐type patients (white arrows). Most patients hosting the new E‐type CTCs (red arrow) and the subject having the E/M‐type CTCs following therapy (black arrow) are toward poor prognosis (mPFS < 9.4 months). (A‐b) CTEC. Three cohorts of prior‐to‐therapy patients possess three distinct baseline CTEC subtypes, including M‐type (6 patients), N‐type null cell (9 patients), and E‐type CTECs (6 patients), are identified with relatively equal proportions at *t*_0_. Newly emerged hybrid E/M‐type CTECs (red, 5 patients) are detected in all three baseline cohorts during therapy (*t*_1‐2_, white arrows). All five patients carrying the *de novo* E/M‐type (red arrow) and a majority of subjects possessing the E‐type post‐therapeutic CTECs (black arrow) exhibit an inferior prognosis. Most patients who had post‐therapeutic aneuploid N‐type null CTECs show a better response to treatment (green arrow, mPFS > 9.4 months). (B) Correlation of different cohorts of patients harboring diverse CTC and CTEC subtypes with disease progression. None of the baseline CTC subtypes (numbers in blue) are significantly relevant to poorer prognosis (PD with new mets) (*P* = 0.652), whereas post‐therapeutic EpCAM^+^ CTCs, regardless of Vim expression or not, significantly correlate with poorer prognosis (*P = 0.037, red). Both baseline Vim^+^ and post‐therapeutic EpCAM^+^ CTECs demonstrate a significant correlation with poorer prognosis, *P = 0.023 and *P = 0.017 (red), respectively. (C) Dichotomized Kaplan–Meier survival analysis. (C‐a) Pretherapeutic patients possessing baseline vimentin^+^ CTECs have a mPFS of 5.0 months compared with 10.1 months in those without vimentin^+^ CTECs (**P = 0.009, log‐rank test). (C‐b) Post‐therapeutic patients having EpCAM^+^ CTECs following therapy show a shorter mPFS of 5.6 months, whereas subjects who have no EpCAM^+^ CTECs reveal a prolonged mPFS of 18.3 months (**P = 0.002, log‐rank test). (C‐c) Compared to a mPFS of 10.1 months in post‐therapeutic patients without EpCAM^+^ CTCs, subjects with detectable EpCAM^+^ CTCs exhibited a reduced mPFS of 5.6 months (*P = 0.017, Breslow test).

Longitudinal analysis of subcategorized cohorts of subjects hosting diverse subtypes of CTCs and CTECs at the designated time intervals during therapy is graphically shown in Sankey diagrams (Fig. 5Aa‐b). The mPFS of 9.4 months in the descending cohort (Fig. 4A, blue line) is taken as the stratification standard for classification of improved (> 9.4 months) or poorer (< 9.4 months) prognosis. The number of patients harboring each subtype of cells is indicated in Fig. 5A,B. As demonstrated in Fig. 5Aa, among 21 recruited patients including both ascending (7 patients) and descending cohorts (14 patients, Fig. 3A, Table 1), three different subtypes of CTCs were identified at baseline (*t*_0_), consisting of EpCAM^−^/Vim^+^ (M‐type, 3 patients), EpCAM^−^/Vim^−^ (nonhematologic aneuploid N‐type null cells, 17 patients), and hybrid EpCAM^+^/Vim^+^ (E/M‐type, 1 patient) CTCs. Following combination therapy, a cohort of patients having the *de novo* subtype of EpCAM^+^/Vim^−^ (E‐type) CTCs emerged (red, *t*_1‐2_, 7 patients). The majority of these patients were subjects containing EpCAM^−^/Vim^−^ N‐type null CTCs at baseline, and a minority possessed M‐type CTCs prior to therapy (white arrows). Most patients who contained the emerged E‐type CTCs (red arrow) and the subject possessing the E/M‐type CTCs (black arrow) following therapy (*t*_1‐2_) were toward poor prognosis (mPFS < 9.4 months). As depicted in Fig. 5Ab, three subtypes of baseline CTECs, including M‐type (6 patients), E‐type (6 patients) and N‐type null CTECs (9 patients), were respectively detected in 21 pretreatment patients with relatively equal proportions at *t*_0_. A new subtype of EpCAM^+^/Vim^+^ CTECs (E/M‐type, red, 5 patients) was detected in all three baseline cohorts following therapy (*t*_1‐2_, white arrows). All patients who had the *de novo* identified subtype of E/M‐type CTECs (red arrow) and a majority of subjects having the E‐type CTECs following therapy (black arrow) exhibited an inferior prognosis. Most patients who had post‐therapeutic aneuploid N‐type null CTECs during therapy showed a better response to treatment (green arrow, mPFS > 9.4 months).

Sankey diagram graphical analysis indicated that patients who had post‐therapeutic EpCAM^+^ CTCs or CTECs independently of vimentin expression showed a poorer prognosis (mPFS < 9.4 months), whereas the majority of those who possessed EpCAM^−^ CTECs following therapy, exhibited an improved survival.

Further quantitative analysis was performed to examine correlations of different cohorts of patients bearing stratified subtypes of CTCs and CTECs with disease progression. As illustrated in Fig. 5B, evaluated patients were classified into two categories: (a) poorer prognosis: PD with new metastasis (newly developed intrapulmonary or distant metastasis), and (b) stable disease (SD) or PD only with enlarged primary lesion but no new metastasis (mets). Three baseline CTC subtypes shown in Fig. 5Aa did not demonstrate significant correlation with patients’ poorer prognosis (*P* = 0.652). Post‐therapeutic CTCs revealed subtypes comprising all possible four combinations of EpCAM and vimentin expression phenotypes. Seven out of eight patients (7/8 = 87.5%) harboring EpCAM^+^ CTCs following therapy, regardless of vimentin expression or not, exhibited a poorer prognosis. Nine of the remaining 13 patients (9/13 = 69.2%) possessing post‐therapeutic EpCAM^−^ CTCs had no new mets at PD. Correlation of post‐therapeutic EpCAM^+^ CTCs with patients’ poorer prognosis was statistically significant (*P = 0.037).

Regarding baseline CTECs, all six patients (100%) possessing M‐type CTECs at baseline had a poorer prognosis. Among the remaining 15 patients who possessed baseline Vim^−^ CTECs, 10 of them (10/15 = 66.7%) showed no new mets at PD. Correlation of positive detection of baseline Vim^+^ CTECs with poorer prognosis was statistically significant (*P = 0.023). Concerning 21 follow‐up patients, 10 of 11 subjects harboring post‐therapeutic EpCAM^+^ CTECs (9/11 = 81.8%) revealed a poorer prognosis regardless of vimentin expression or not. Among remaining 10 patients bearing EpCAM^−^ CTECs, nine subjects (8/10 = 80%) did not have new mets at PD. Correlation between the existence of post‐therapeutic EpCAM^+^ CTECs and the poorer prognosis was statistically significant (*P = 0.017).

The above analyses demonstrated that EpCAM^+^ CTCs and CTECs as well as Vim^+^ CTECs were relevant to patients’ poor response to treatment. The observation was further confirmed by Cox regression analysis for risk stratification. As revealed in Fig. S1, in comparison with other variates, positively detected baseline and post‐therapeutic CTECs expressing vimentin, post‐therapeutic CTCs and CTECs expressing EpCAM were the significant cellular univariable risk factors for PFS (*P* < 0.05, red), showing that subjects bearing baseline Vim^−^ CTECs (HR = 0.266, 95% CI: 0.091–0.777), post‐therapeutic Vim^−^ CTECs (HR = 0.165, 95% CI: 0.044–0.621), post‐therapeutic EpCAM^−^ CTECs (HR = 0.210, 95% CI: 0.072–0.615) or EpCAM^−^ CTCs (HR = 0.268, 95% CI: 0.089–0.808), had a lower risk (HR < 1) for a rapid disease progression compared to cohorts containing Vim^+^ or EpCAM^+^ cells. Accordingly, dichotomized Kaplan–Meier survival analysis was performed to further investigate how Vim^+^ or EpCAM^+^ CTCs and CTECs in all recruited NSCLC patients responded to bevacizumab combination therapy (Fig. 5C). As depicted in Fig. 5Ca, pretherapeutic subjects who had baseline Vim^+^ CTECs (*N* = 6) showed a reduced mPFS of 5.0 months (95% CI: 0–10.4 months) compared to 10.1 months (95% CI: 1.6–30.6 months) in those without baseline Vim^+^ CTECs (*N* = 19, **P = 0.009, log‐rank test). Although Cox regression analysis revealed post‐therapeutic Vim^+^ CTECs as a significant univariable risk factor, Kaplan–Meier survival analysis did not show a significant difference on mPFS between post‐therapeutic Vim^+^ and Vim^−^ CTECs cohorts (*P* = 0.132), possibly due to small sample size. Further K‐M survival analysis was performed to examine post‐therapeutic EpCAM^+^ *vs* EpCAM^−^ cohorts, regardless of vimentin expression or not. As depicted in Fig. 5Cb, patients possessing EpCAM^+^ CTECs following therapy (*N* = 11) showed a reduced mPFS of 5.6 months (95% CI: 1.6–9.4 months), whereas those who had no EpCAM^+^ CTECs (*N* = 14) revealed a prolonged mPFS of 18.3 months (95% CI: 2.4–34.2 months) (**P = 0.002, log‐rank test). Regarding post‐therapeutic CTCs, as demonstrated in Fig. 5Cc, subjects having EpCAM^+^ CTCs (*N* = 9) exhibited a mPFS of 5.6 months (95% CI: 3.8–7.4 months) compared to 10.1 months (95% CI: 3.2–17.0 months) in patients without post‐therapeutic EpCAM^+^ CTCs (*N* = 16, *P = 0.017, Breslow test).

Additional multistrata Kaplan–Meier survival analyses in Fig. S2 showed patients who respectively had diverse subtypes of CTECs at baseline displayed a mPFS of 16.1 (N‐type, null cell) > 9.2 (E‐type) > 5.0 months (M‐type), *P = 0.027 (Fig. S2a, log‐rank test), suggesting that baseline M‐type CTECs significantly correlated with poorer prognosis. Moreover, subjects hosting disparate subtypes of post‐therapeutic CTECs revealed a mPFS of 18.3 (N‐type, null cell) > 9.2 (M‐type) > 6.5 (E‐type) > 3.0 months (E/M‐type), **P = 0.004 (Fig. S2b, log‐rank test), implying that patients who acquired E/M‐type CTECs following therapy had the most inferior outcome, which kept in accordance with the Sankey analysis demonstrating that entire cohorts possessing post‐therapeutic EpCAM^+^/Vim^+^ E/M‐type CTCs or CTECs were toward poor prognosis. Cohorts having either pre‐ or post‐therapeutic EpCAM^−^/Vim^−^ N‐type null CTECs exhibited a better response to treatment, showing an improved longest mPFS.

## 4 Discussion

The specific relevance of TECs in circulation (CTECs) to the vasculature of malignant tumors remains a challenging topic [44]. Aneuploidy is a hallmark of malignancy that drives lethal progression in cancer cells, showing the degree of aneuploidy is proportional to the grade of malignancy of neoplastic cells, the higher the degree of aneuploidy, the higher frequency of *KRAS* and *TP53* mutations, and the higher the malignancy grade, as well as the adverse prognosis [40, 45]. In opposition to conventional diploid CECs, aneuploid TECs [5] in circulation, that is, CTECs [11, 12, 46], are more relevant to tumor neovascularization and cancer metastasis [6, 12, 47]. In this exploratory prospective study, guided by Cox proportional hazards regression analysis for risk stratification, we specifically investigated whether the identified cell‐based significant univariable risk factors of quantitative variation trend of aneuploid CTCs and CD31^+^ CTECs as well as positive detection of their subtypes expressing EpCAM and/or vimentin may function as surrogate biomarkers to predict and correlate with response to anti‐angiogenic therapy.

In contrast to markedly reduced overall circulating ECs in chemotherapeutic breast cancer patients at PD [48], the present study demonstrated that an upward trend in terms of a concurrent increase in a total number of post‐therapeutic aneuploid CTECs and CTCs in the ascending cohort significantly correlated with a shortened mPFS and mOS (Fig. 4B). A downward trend showing a decrease in the number of total aneuploid CTECs and CTCs in the descending cohort at *t*_2_ correlated with a prolonged mPFS and mOS. The obtained results implied that both the total amount of aneuploid CTECs and CTCs detected after four‐to‐six treatment cycles (*t*_2_) may be able to function as an indicative biomarker to timely index patients’ response to combination regimen.

Aside from enumeration of total CTCs and CTECs alone, further *in situ* phenotypic and karyotypic characterization performed by iFISH was applied to investigate whether and how the prognosticators vimentin [28, 29], EpCAM [21, 25, 26], and aneuploidy [40] in the specific subtypes of CTCs as well as CTECs correlated with patients’ prognosis. In line with vimentin [29], an accelerator for tumor growth, invasion, progression, and metastasis, as well as an independent prognosticator for poor prognosis and survival in various epithelial cancer patients [28, 29], the current study demonstrated that only the specific subtype of baseline Vim^+^ CTECs with multiploid chr8 exhibited a predictive value for patients’ poor prognosis, showing that all patients harboring this subtype of CTECs prior to treatment had new mets at PD (Fig. 5B) and a shortened mPFS (Fig. 5Ca). The obtained results are in accordance with our previously published study indicating that baseline Vim^+^ aneuploid circulating rare cells including CD31^+^ CTECs significantly correlated with poor prognosis as well as distant hepatic metastasis in advanced lung cancer patients [49]. Our results on CTECs were in conformity with studies published by others on bevacizumab‐treated colorectal cancer patients showing that a high baseline number of CECs was a strong independent prognosticator for worsened PFS [50]. The present study suggested that baseline Vim^+^ M‐type multiploid CTECs might possess clinical utility in predicting NSCLC patients’ poor response to anti‐angiogenic bevacizumab.

As illustrated in Fig. 5A,B, *de novo* identified post‐therapeutic EpCAM^+^ E‐type CTCs and EpCAM^+^/Vim^+^ E/M‐type CTECs were related to poor prognosis. The E/M phenotype is in line with a previously published study showing that E/M‐type breast CTCs, harboring enhanced epithelial cell adhesion and extravasation capability, represent more aggressive cancer cells with highest metastasis ability [26]. The intermediate hybrid E/M‐type cancer cells were found to present high plasticity to adapt to secondary metastatic sites and a high potential to constitute tumor stem cells, resulting in enhanced metastasis formation in varieties of cancer patients [42, 51]. Existence of EpCAM^+^_S_CTCs*^di^* and EpCAM^+^_L_CTECs*^multi^* following 4–6 cycles of therapy (*t*_2_) in the ascending cohort, as revealed in Table 2, indicates that these specific subtypes of cells might develop resistance to the combination therapy [52, 53]. This suggests that detecting EpCAM^+^ CTCs and CTECs in post‐therapeutic lung cancer patients is of particular clinical utility, in terms of timely detecting emerging therapeutic resistance and appraising patients’ poor response to combination therapy. The conclusion was supported by Kaplan–Meier survival analysis, demonstrating that post‐therapeutic EpCAM^+^ CTECs and CTCs significantly correlated with patients’ reduced mPFS (Fig. 5Cb‐c). In particular, the EpCAM^+^/Vim^+^ E/M‐type CTEC cohort had the shortest mPFS of 3.0 months (Fig. S2b). Obtained results suggested that the post‐therapeutic EpCAM^+^ CTECs and CTCs might be able to function as prognosticators for poor outcome.

It is interesting to indicate although six out of 21 patients had baseline EpCAM^+^ E‐type CTECs detected, no E‐type CTC was detected at baseline. Most baseline CTCs were aneuploid EpCAM^−^/vimentin^−^ N‐type null cells. Frequently reported low or absence of EpCAM on NSCLC cells [54, 55] and active EMT that results in down‐regulation of EpCAM on CTCs [20, 21, 22, 56], may account for the existence of predominant null CTCs in pretherapeutic patients in this study.

The Sankey diagram analysis provides a convenient graphical approach for retrospectively tracing and analyzing how different cohorts of patients are relevant to each other and to correlate with the ultimate outcome throughout therapy. Sankey analysis (Fig. 5A) displayed how diverse cohorts of subjects carrying prognosis‐relevant CTC and CTEC subtypes (including newly arisen subtypes) were relevant to each other during therapy, suggesting that proliferation of diverse subclones of disparate CTC and CTEC subtypes or longitudinal subtype transition may occur along with therapeutically stressed tumor progression and treatment process. Future *in vivo* studies performed on the potential lung cancer metastatic PDX (mPDX) models [52] treated with bevacizumab will help uncover cellular and molecular mechanisms regarding how disparate subclones‐derived CTC and CTEC subtypes are related and regulated, and whether EMT as well as EndoMT may play a role in creation of EpCAM^+^ and/or Vim^+^ CTCs and CTECs in cancer patients during therapy.

We previously reported that about 66% healthy subjects (*n* = 47) had aneuploid EpCAM^−^/vimentin^−^ null ECs detected in peripheral blood with an average of 2.8 cells compared with 100% in all carcinoma patients (*n* = 133) with an average of 8.8 cells, and none of EpCAM^+^ or Vim^+^ circulating CD31^+^ cells were detected in healthy donors [11]. It cannot be ruled out that some abnormal aneuploid EpCAM^−^/Vim^−^ circulating ECs observed in healthy donors might also exist in cancer patients. Following the emergence of clinical utilities of EpCAM^+^ and/or vimentin^+^ aneuploid CTEC subtypes revealed in this study, several intriguing questions require further single cell‐based molecular unraveling, such as whether aneuploid CECs in healthy subjects are precancerous‐related cells and can be homeostatically depleted by immune scavengers of the host defense system [57], whether aneuploid null CECs observed in healthy subjects may also exist in cancer patients and potentially relevant to patients’ response to therapy, whether those aneuploid null CECs in healthy donors and different subtypes of aneuploid CTECs in cancer patients are molecularly related to each other or differentially express TEMs [12, 13], etc. Answering these questions will shed light on further uncovering how aneuploid CTECs function in carcinoma patients.

The present study reported the clinical relevance of CTCs and CTECs, particularly EpCAM^+^ and/or Vim^+^ cells, in bevacizumab‐treated advanced NSCLC patients. The strategy described in this study provided a novel and meaningful alternative approach adequate for future prospective and comprehensive studies. It has to be indicated that cohort categorization and relevant Cox analyses may not be perfect in this study due to the limited sample size, it is necessary to carry out expanded studies that implement a stratification of a large cohort of lung cancer or other carcinoma patients. Such studies will help further validate the robustness of clinical utilities of CTCs and CTECs, and unravel essential insights regarding how aneuploid CTCs and CTECs interplay in tumorigenesis, tumor neovascularization, cancer metastasis, and response to anti‐angiogenesis in combination with chemotherapy, tyrosine kinase inhibitors including erlotinib targeting the *EGFR*‐L858R activating mutation [58, 59], and the novel third‐generation TKI osimertinib (Tagrisso^®^) targeting the *EGFR*‐T790M resistance mutation [60], as well as immune checkpoint blockade immunotherapy [17, 61].

## 5 Conclusions

The present study provides proof of the concept showing that aneuploid CD31^−^ CTCs and CD31^+^ CTECs may function as a pair of cellular circulating tumor biomarkers in predicting and prognosticating NSCLC patients subjected to anti‐angiogenic combination therapy. An upward trend in terms of an increase in total quantified post‐therapeutic CTC and CTEC numbers correlates with patients’ poor response to bevacizumab, showing a reduced mPFS and mOS. Existence of the specific subtype of vimentin^+^ mesenchymal CTECs at baseline, correlating with poor response to anti‐angiogenic therapy, might be an adverse biomarker in appropriately selecting eligible subjects. Furthermore, compared to conventional clinical imaging evaluation on tumor mass, positively detected post‐therapeutic EpCAM^+^ CTECs and CTCs, particularly *de novo* EpCAM^+^/Vim^+^ E/M‐type CTECs, may be indicative biomarkers in terms of timely indexing therapeutic efficacy and detecting emerging resistance to treatment. A majority of patients who displayed either pre‐ or post‐therapeutic aneuploid EpCAM^−^/Vim^−^ N‐type null CTECs during therapy exhibited a better response to the combination regimen. In contrast to CD31^−^ CTCs, which are highly heterogeneous in both cell sizes and degrees of aneuploidy, the main population of CD31^+^ CTECs are homogeneous, large multiploid cells (>WBC size, ≥pentasomy 8, _L_CTECs*^multi^*).

## Conflict of interest

i•FISH^®^ is the registered trademarks of Cytelligen. Dr. Peter P. Lin is the president at Cytelligen. None of authors owns Cytelligen's stock shares. No additional COI to be disclosed.

## Author contributions

TZ contributed conceptualization, validation, formal analysis, investigation, resources, funding acquisition, and writing—original draft; LZ contributed conceptualization, methodology, validation, formal analysis, investigation, data curation, funding acquisition, writing—original draft, visualization, and funding acquisition; YG contributed validation, formal analysis, investigation, resources, and project administration; YW contributed investigation and data curation; YL contributed investigation and data curation; HZ contributed conceptualization and investigation; QW contributed conceptualization and investigation; FH contributed conceptualization and investigation; JL contributed conceptualization and investigation; JT contributed formal analysis and software; DDW contributed methodology and validation; OG contributed writing—original draft, review, editing, and revising manuscript critically for important intellectual content; PPL contributed conceptualization, visualization, writing—original draft, review, and editing; BL contributed conceptualization, resources, and supervision. All the authors read and approved the final version of manuscript.

### Peer Review

The peer review history for this article is available at https://publons.com/publon/10.1002/1878‐0261.13092.

## Supporting information


**Fig. S1**. Risk stratification by Co*x* proportional hazards regression model analysis. Risk factors including non‐cell‐based gender, age, staging, and cell‐based quantitative variation trend of CTCs or CTECs (ascending *vs* descending cohort), positive detection of EpCAM^+^ CTCs or CTECs either prior to or post‐therapy, vimentin^+^ CTCs or CTECs either prior to or post‐therapy, are analyzed by the Cox regression analysis. *Results of Cox regression analysis performed on ascending vs descending cohorts classified by CTECs are identical to CTCs. Significant univariable risk factors (*P* < 0.05) are indicated in red font. HR: hazard ratio, HR > 1: higher risk, HR < 1: lower risk; n/a: not available, no death occurred.

**Fig. S2**. Multistrata Kaplan–Meier survival analyses. (a) Baseline CTECs: patients with EpCAM^−^/Vim^+^ M‐type CTECs have a mPFS of 5.0 months compared to 9.2 months and 16.1 months in the cohorts of EpCAM^+^/Vim^−^ E‐type and EpCAM^−^/Vim^−^ null cell, *P = 0.027 (log‐rank test). (b) Post‐therapeutic CTECs: patients having EpCAM^+^/Vim^+^ (hybrid E/M‐type) CTECs reveal the shortest mPFS of 3.0 months. The other three cohorts including EpCAM^+^/Vim^−^ (E‐type), EpCAM^−^/Vim^+^ (M‐type) and EpCAM^−^/Vim^−^ null CTECs show the mPFS of 6.5, 9.2 and 18.3 months, respectively, **P = 0.004 (log‐rank test). Cohorts possessing either pretherapeutic or post‐therapeutic EpCAM^−^/Vim^−^ null CTECs have a better response to treatment, displaying a prolonged mPFS.

## Data Availability

Software used in this manuscript are publicly available, mentioned in the methods section. The datasets used and/or analyzed during the current study are available from the corresponding author on reasonable request.
