# Supplementary figures and images for: Role of aneuploid circulating tumor cells and CD31+ circulating tumor endothelial cells in predicting and monitoring anti‐angiogenic therapy efficacy in advanced NSCLC
Source: Mol Oncol. 2021 Sep 12;15(11):2891–909. doi: 10.1002/1878-0261.13092 (PMC8564645; doi:10.1002/1878-0261.13092)

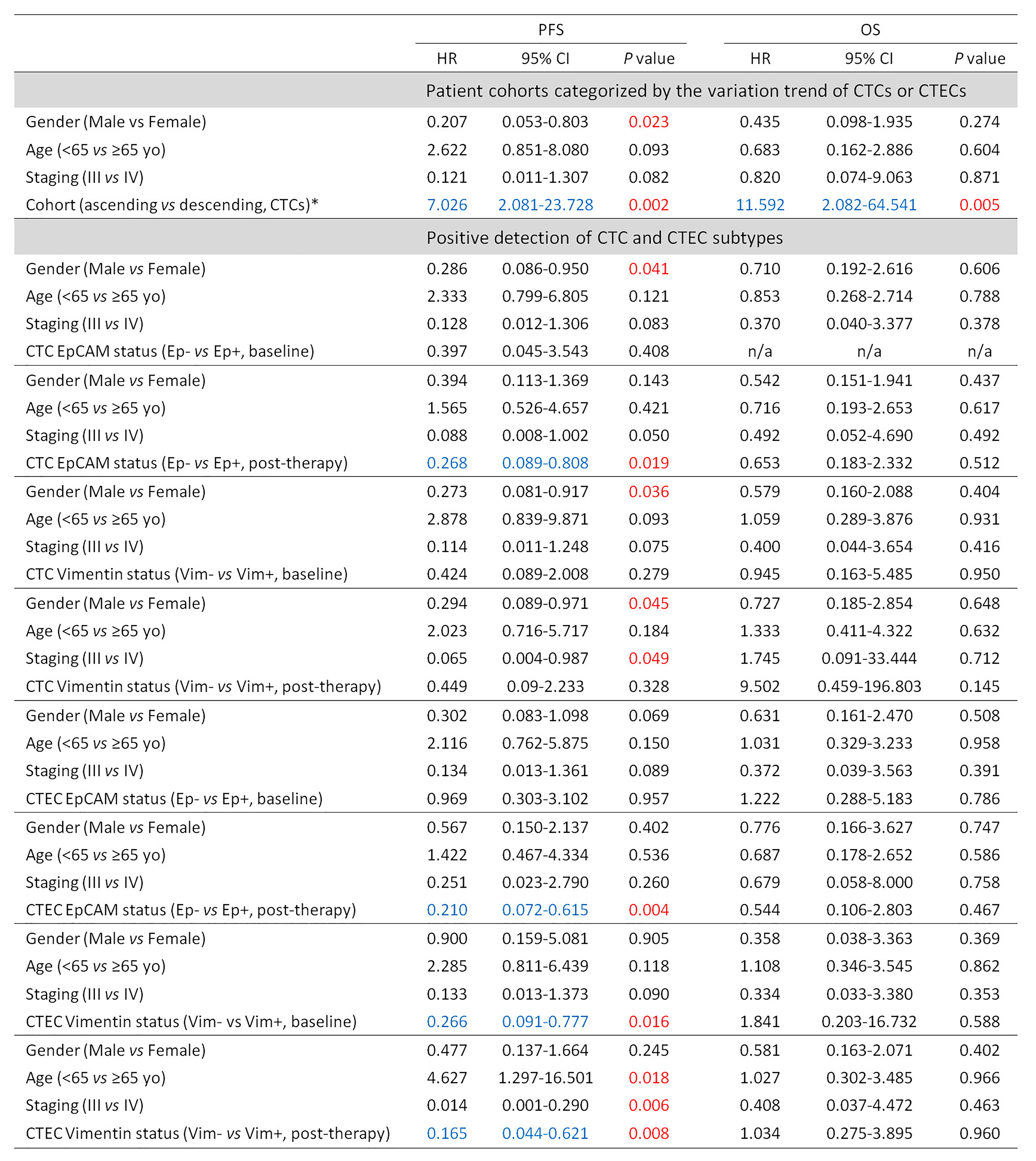

Supplement: Supplementary file 1 — Fig. S1. Risk stratification by Cox proportional hazards regression model analysis. Risk factors including non‐cell‐based gender, age, staging, and cell‐based quantitative variation trend of CTCs or CTECs (ascending vs descending cohort), positive detection of EpCAM+ CTCs or CTECs either prior to or post‐therapy, vimentin+ CTCs or CTECs either prior to or post‐therapy, are analyzed by the Cox regression analysis. *Results of Cox regression analysis performed on ascending vs descending cohorts classified by CTECs are identical to CTCs. Significant univariable risk factors (P < 0.05) are indicated in red font. HR: hazard ratio, HR > 1: higher risk, HR < 1: lower risk; n/a: not available, no death occurred. [file MOL2-15-2891-s002.jpg]

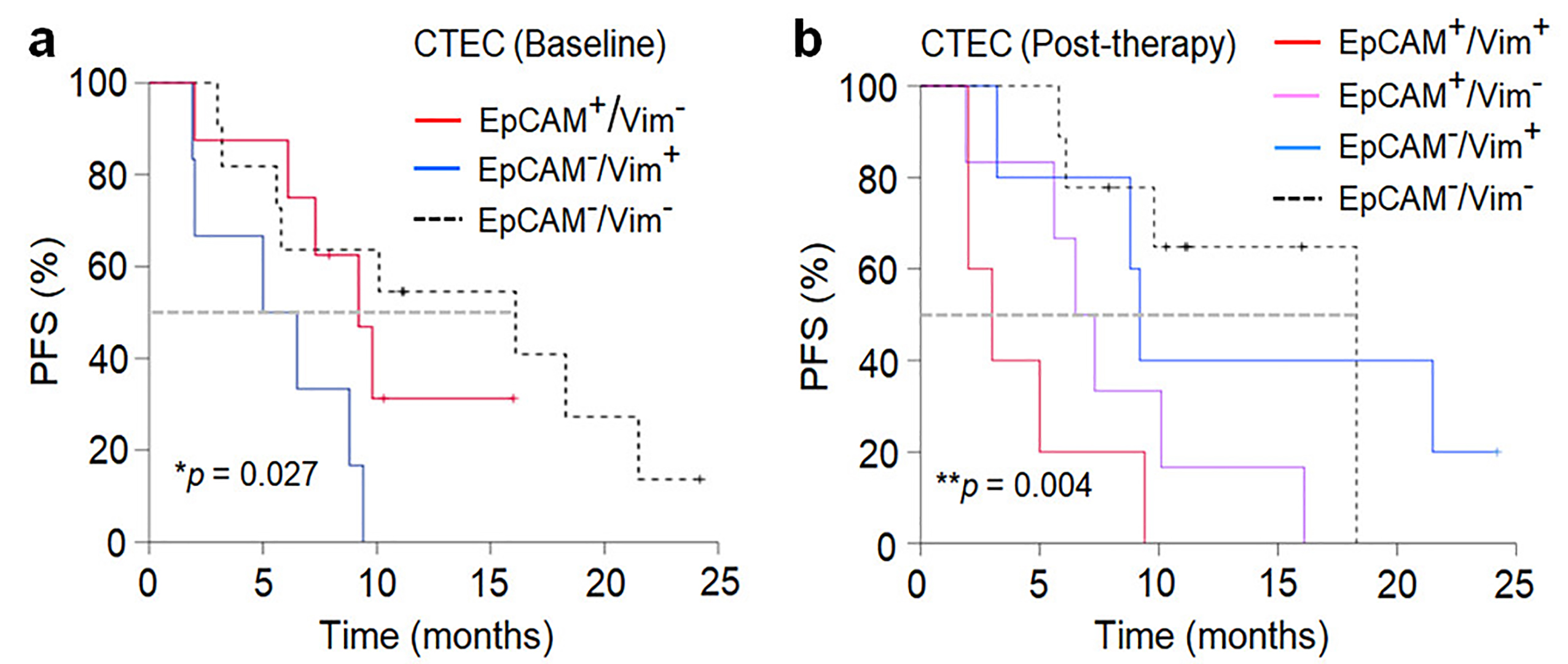

Supplement: Supplementary file 2 — Fig. S2. Multistrata Kaplan–Meier survival analyses. (a) Baseline CTECs: patients with EpCAM−/Vim+ M‐type CTECs have a mPFS of 5.0 months compared to 9.2 months and 16.1 months in the cohorts of EpCAM+/Vim− E‐type and EpCAM−/Vim− null cell, *P = 0.027 (log‐rank test). (b) Post‐therapeutic CTECs: patients having EpCAM+/Vim+ (hybrid E/M‐type) CTECs reveal the shortest mPFS of 3.0 months. The other three cohorts including EpCAM+/Vim− (E‐type), EpCAM−/Vim+ (M‐type) and EpCAM−/Vim− null CTECs show the mPFS of 6.5, 9.2 and 18.3 months, respectively, **P = 0.004 (log‐rank test). Cohorts possessing either pretherapeutic or post‐therapeutic EpCAM−/Vim− null CTECs have a better response to treatment, displaying a prolonged mPFS. [file MOL2-15-2891-s001.jpg]
